# The relationship between obstructive sleep apnea and obesity hypoventilation syndrome: a systematic review and meta-analysis

**DOI:** 10.18632/oncotarget.21450

**Published:** 2017-10-03

**Authors:** Chaoling Liu, Mao-Sheng Chen, Hui Yu

**Affiliations:** ^1^ Respiratory Department, Guangdong Provincial Hospital of Chinese Medicine & the 2nd Clinical College of Guangzhou University of Chinese Medicine, Guangzhou, 510120, China; ^2^ Division of Chest Pain Center, Guangdong Provincial Hospital of Chinese Medicine & the 2nd Clinical College of Guangzhou University of Chinese Medicine, Guangzhou, 510120, China

**Keywords:** obesity hypoventilation syndrome, obstructive sleep apnea, obesity, hypercapnia, hypoventilation

## Abstract

Obstructive Sleep Apnea and Obesity Hypoventilation Syndrome are two similar diseases. Obstructive Sleep Apnea has been receiving more and more attention while the diagnostic rate of Obesity Hypoventilation Syndrome is not high. Few studies directly evaluated the relationship between them. We systematically analyzed the relevance of the two diseases. MEDLINE^®^, EMBASE^®^ and the Cochrane Library were carried out to find studies until May 2017. Pooled mean difference and 95% confidence interval were calculated to evaluate the value of clinical and physiologic variables in the prediction of Obesity Hypoventilation Syndrome. 9 Studies (*n* = 2085) fulfilled the predefined selection criteria. Totally 575 patients (28%) with Obesity Hypoventilation Syndrome were diagnosed from 2085 Obstructive Sleep Apnea patients. Among clearly diagnosed Obstructive Sleep Apnea patients, higher Body Mass Index levels(mean difference:4.72 kg/m^2^; 95% confidence interval: 4.26 to 5.17; *p* < 0.00001), higher Apnea-Hypopnea Index (mean difference: 8.36; 95% confidence interval: *−*3.88 to −2.84; *p* < 0.00001), greater neck circumference (mean difference:1.01; 95% confidence interval: 0.10 to 1.92; *p* = 0.03) and lower percent predicted FEV1 (mean difference:−10.28; 95% confidence interval:−11.33 to −9.22; *p* < 0.00001)were associated with the occurrence with obesity hypoventilation syndrome. We should be highly skeptical of obesity hypoventilation syndrome in Obstructive Sleep Apnea patients with these factors as early identification and appropriate treatment can improve prognosis.

## INTRODUCTION

Obesity hypoventilation syndrome (OHS) is characterized by obesity, chronic daytime hypercapnia, and sleep-disordered breathing in the absence of other known causes of hypercapnia [1]. It also can be called Pickwikian Syndrome which was firstly proposed by Buewell [2]. Similar to OHS, obstructive sleep apnea (OSA) is also a kind of disease relevant with obesity. The prevalence of OSA in adult male is about 3% to 7%, while the number is 2% to 5% in adult female in the U.S [3]. It is estimated that the incidence of OHS patients in Europe and the United States was between 0.3% to 0.4% [4, 5]. There are a lot of similarities between them in pathogenesis, pathophysiology and clinical manifestations. It is important for clinical doctors to differentiate OHS and OSA when making a diagnosis. OHS is an exclusionary diagnosis while OSA can be diagnosed by polysomnography.

Soylu, A.C. et al. showed that the risk of OSA is significantly higher in men with neck circumference greater than 40 cm and in women with neck circumference greater than 36 cm [6]. It is mainly because that too much fat accumulates in the patients’ neck, leading to significantly reduced pharyngeal cross-sectional area and weakened pharyngeal muscle support force. Brennick, M.J. et al. thought upper airway is easy to be obstructed for these reasons, especially in the process of sleep [7]. Vgontzas et al. found that subcutaneous fat of OSA patients was not necessarily different from the overall fat, but the accumulation of visceral fat increased significantly [8]. Considering that the accumulation of visceral fat will limit the movement of the diaphragm, chest wall will decrease as a result. Furthermore, the lung volume would be smaller so that the expansion is limited which leads to hypoxia in patients especially when lying. OHS is also influenced by obesity. Koenig et al. reported that respiratory compliance of OHS patients could decrease to 56% –63% of normal people [9]. In addition, high level of visceral fat accumulation in OHS patients was common, which was the same as neck fat accumulation level. Fat accumulation are likely to lead to upper airway obstruction in OHS patients when they are in the sitting position and supine position, which will undoubtedly make OHS patients’ daytime hypoxemia and hypercapnia worse.

In the present study, 42% of the OHS patients were admitted with acute hypercapnic respiratory failure [10]. Patients may be misdiagnosed frequently as having chronic obstructive pulmonary disease (COPD) or chronic heart disease, resulting in delays in receiving the appropriate treatment, which illustrate that there is a lack of awareness of OHS in clinical work nowadays. The mortality was 9% in obese patients without daytime hypercapnia while the mortality was up to 23% in the OHS group at 18 months following hospital discharge [11]. Studies revealed that OHS patients could have higher health-care expenses [12] poorer prognoses, and higher risks of hospitalization and death compared with pure OSA patients [10, 13].

Therefore, it is particularly important to clinically identify OSA patients with OHS. The purpose of our meta-analysis is to assess the prevalence, clinical characteristics, predictors of OHS in OSA patients and identify the relationship between OHS and OSA patients.

## MATERIALS AND METHODS

### Literature-search strategy

Our study strictly complies with the guidelines of the meta-analysis of observational studies in epidemiology group (MOOSE) [14]. A literature search was performed in May 2017 without restriction to regions, publication types, or languages. A comprehensive systematic search of MEDLINE^®^, EMBASE^®^ and Cochrane Library were carried out to find relevant studies until May 2017. The following MeSH terms and their combinations were searched in [Title/Abstract]: obstructive sleep apnea OR OSAHS OR OSA, obesity hypoventilation syndrome OR OHS. The related articles function was also used to broaden the search, and the computer search was supplemented with manual searches of the reference lists of all retrieved studies, review articles, and conference abstracts.

### Inclusion and exclusion criteria

All available comparative studies (cohort or case–control studies) that compared OHS patients with OSA and pure OSA patients, and that had at least one of the quantitative outcomes mentioned in the paper were included. Editorials, letters to the editor, reviews, case reports, and animal experimental studies were excluded.

### Identification of studies

We restricted our search to studies published in English. Abstracts and titles of related articles were initially scanned by a reviewer. Potentially relevant articles were then considered by at least 2 independent reviewers. Any disagreement was resolved by the adjudicating senior authors. Two reviewers agreed on the inclusionary or exclusionary status of 90% of the reviewed studies. Full texts of the selected articles were then screened by all authors for inclusion.

### Quality assessment and data extraction

The methodological quality of observational studies was assessed by the modified Newcastle-Ottawa scale(NOS) [15, 16]. They’re listed in Supplementary Table 1. A score of 0–9 (allocated as stars) was allocated to each study: patient selection, comparability of the groups and assessment of outcomes of interest. Observational studies achieving six or more stars were considered to be of high quality. Two blinded reviewers independently used a standardized data-extraction to determine whether the paper was appropriate or not.

We extracted data including the lead author's last name, the publication year, and the origin of the studied population, the study design, the characteristics of the studied population (sample size, age, gender). The following risk factors were collected from the studies, for both the OSA and OHS+OSA groups: body mass index (BMI); apnea-hypopnea index (AHI); percent predicted FEV1 (FEV1%); percent predicted vital capacity (VC%); percent predicted FEV1/FVC (FEV1/FVC%); mean overnight pulse oximetric saturation (mean SpO_2_); Neck circumference, Waist/hip ratio.

### Statistical analysis

Our meta-analysis was performed using Review Manager 5.2 (Cochrane Collaboration, Oxford, UK). The weighted mean difference (WMD) and odds ratio(OR) were used to compare continuous and dichotomous variables, respectively. All results were reported with 95% confidence intervals (CIs). The significance of pooled mean difference (MD) was tested by *z*-test (*P* < 0.05 was considered significant). Heterogeneity was evaluated with Cochran's Q statistic and quality by *I^2^* statistic. We premeditated that mild heterogeneity might be less than 30 percent of the variability in point estimates and the values of I^2^ exceeding 50% might expressed as significant heterogeneity [17]. The random-effects model was used if there was heterogeneity between studies; otherwise, the fixed-effects model was used. To explore sources of heterogeneity, we performed several sensitivity analyses. Publication bias was also evaluated by inspecting funnel plots. The data conformed to each test that was used to analyze them.

## RESULTS

### Search results

Supplementary Table 4 provides details on how an initial search that yielded 470 potential abstracts was reduced to 9 studies that were included in the meta-analysis (*n* = 2085). 9 observational studies [18–25] were finally fulfilled the predefined inclusion criteria which included in our meta-analysis (Supplementary Table 3). As a result, 2085 patients were involved in our analysis: 1510 patients in OSA group and 575 patients in OSA+OHS group.

### Quality assessment and studies’ characteristics

The NOS for assessing the quality of the 9 studies is shown in Supplementary Table 1. and the scores ranged from 5–8. All the studies were published in the last 15 years, with one exception [18]. 3 studies [18, 20] were performed in white populations, and 2 studies [19, 23] (*n* = 334,16% of total sample) were performed in Japanese patients, the rest studies were from UAE and Turkey respectively. Most of the studies were cohort studies, with the exception of three case-control studies (*n* = 936; 45% of total sample). One study [19] did not provide information about the gender of patients. The majority of patients were men (*n* = 1935; 93%). 5 studies [19, 21, 23–25] clearly identified all the patients as having BMI > 30 kg/m^2^, and the remaining 4 studies [16, 28, 20] either reported patients with a broader range of BMI or the BMI range was unclear. Various cutoffs of AHI were used in the studies to define OSA. Six studies [20–24] defined OSA by using an AHI > 5; one studies [18] used an AHI > 10; and one study [19] used an AHI > 20 to define OSA. One study [25] did not report an AHI cutoff for defining OSA. Weighted means and percentages of patients with and without OHS per risk factor are shown in Supplementary Table 2. Statistical heterogeneity of the effects among studies was present for most factors (*p* < 0.05), with the exception of age (*p* = 0.62), gender (*P* = 0.29), FEV1/FVC (*P* = 0,27) and Waist/hip ratio (*p* = 0.64).

### Outcomes

#### BMI

**Figure 1:**
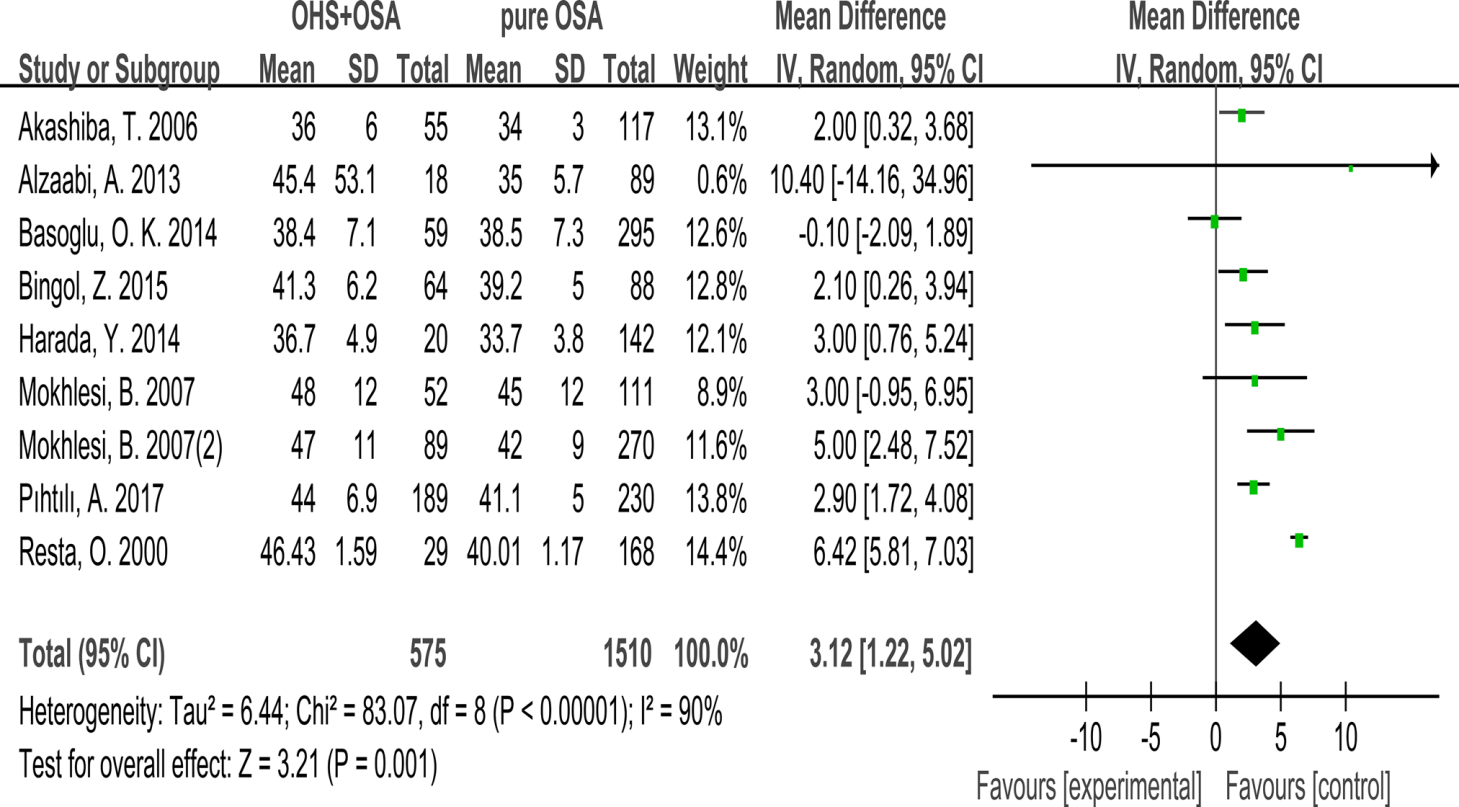
Forest plots of the association between BMI and OHS

9 studies evaluated the association between BMI and OHS.575 patients diagnosed with OSA+OHS and 1510 patients diagnosed with pure OSA were included in the study. It was significantly higher in OHS group. (MD:4.72 kg/m^2^; 95% CI: 4.26 to 5.17; *p* = 0.001) (Figure 1).

### Neck circumference

**Figure 2:**
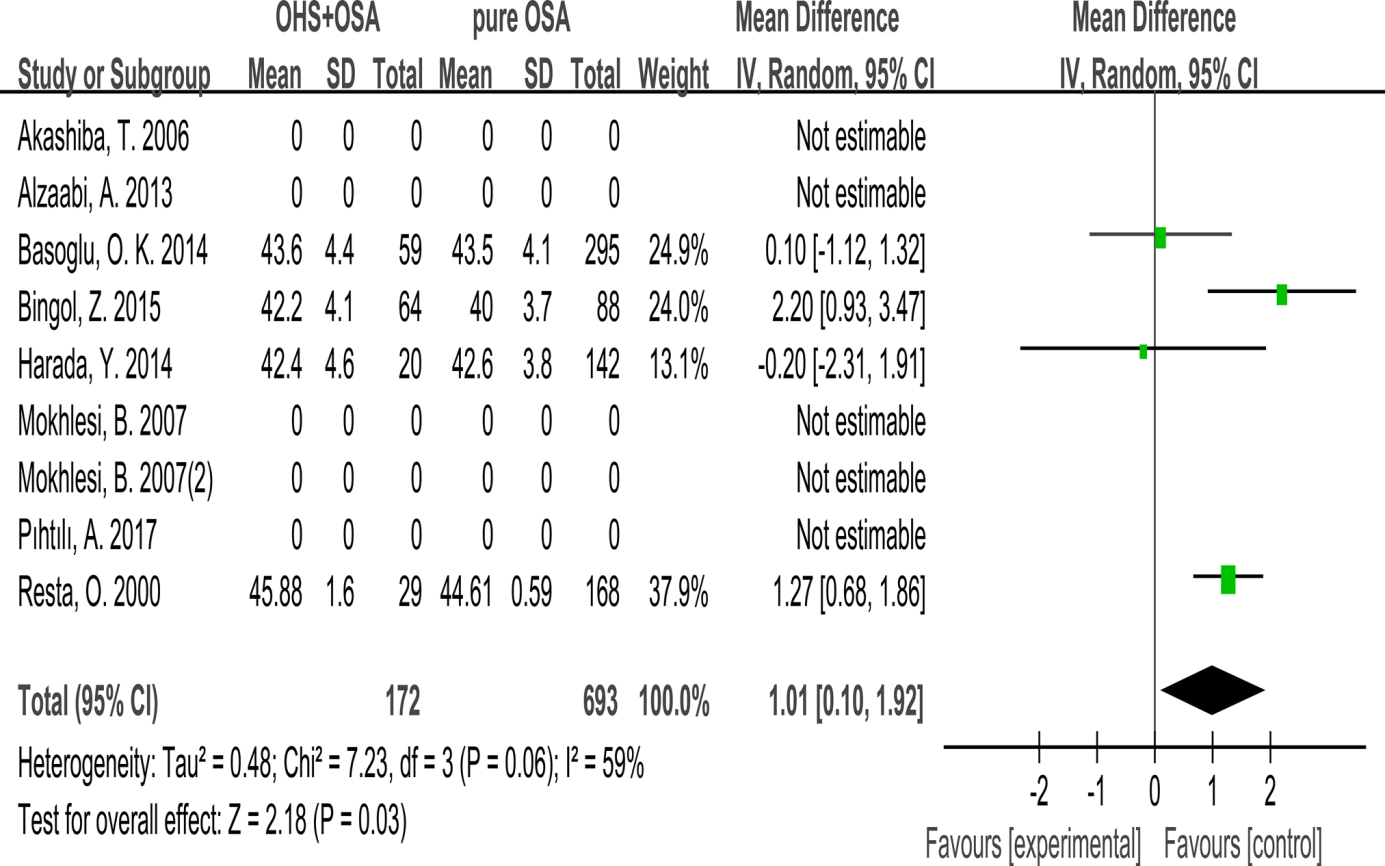
Forest plots of the association between Neck circumference and OHS

Only 4 studies evaluated the association between neck circumference with OHS. It showed the neck circumference was significantly greater in the OHS group than the pure OSA group. (MD:1.01; 95% CI: 0.10 to 1.92; *p* = 0.03) (Figure 2).

### Waist/hip ratio

**Figure 3:**
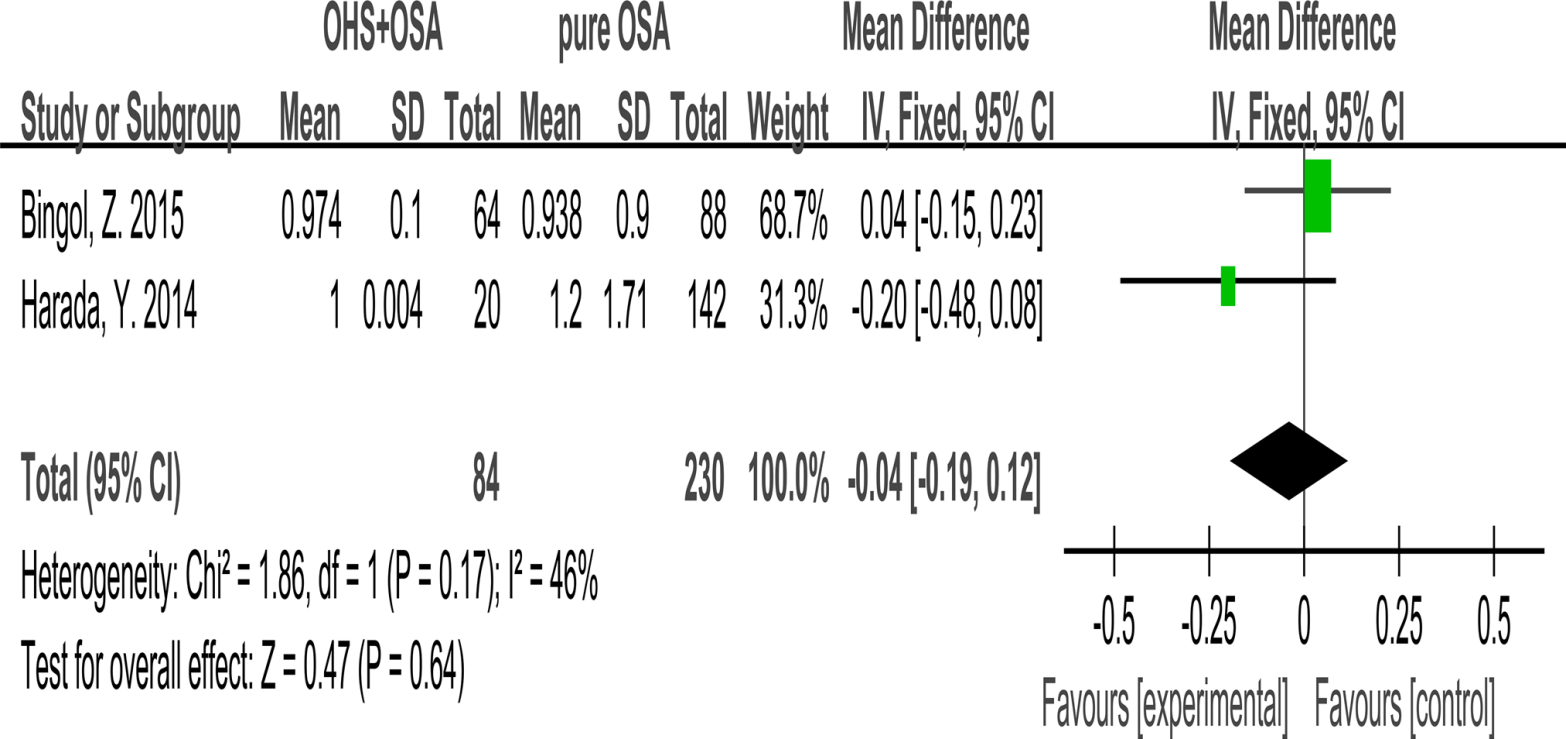
Forest plots of the association between Waist/hip ratio and OHS

2 studies evaluated the association between Waist/hip ratio with OHS. It showed the Waist/hip ratio was not different between the OHS group and the pure OSA group. (MD:–0.04; 95% CI: −0.19 to 0.12; *p* = 0.64) (Figure 3).

### FEV1/FVC

**Figure 4:**
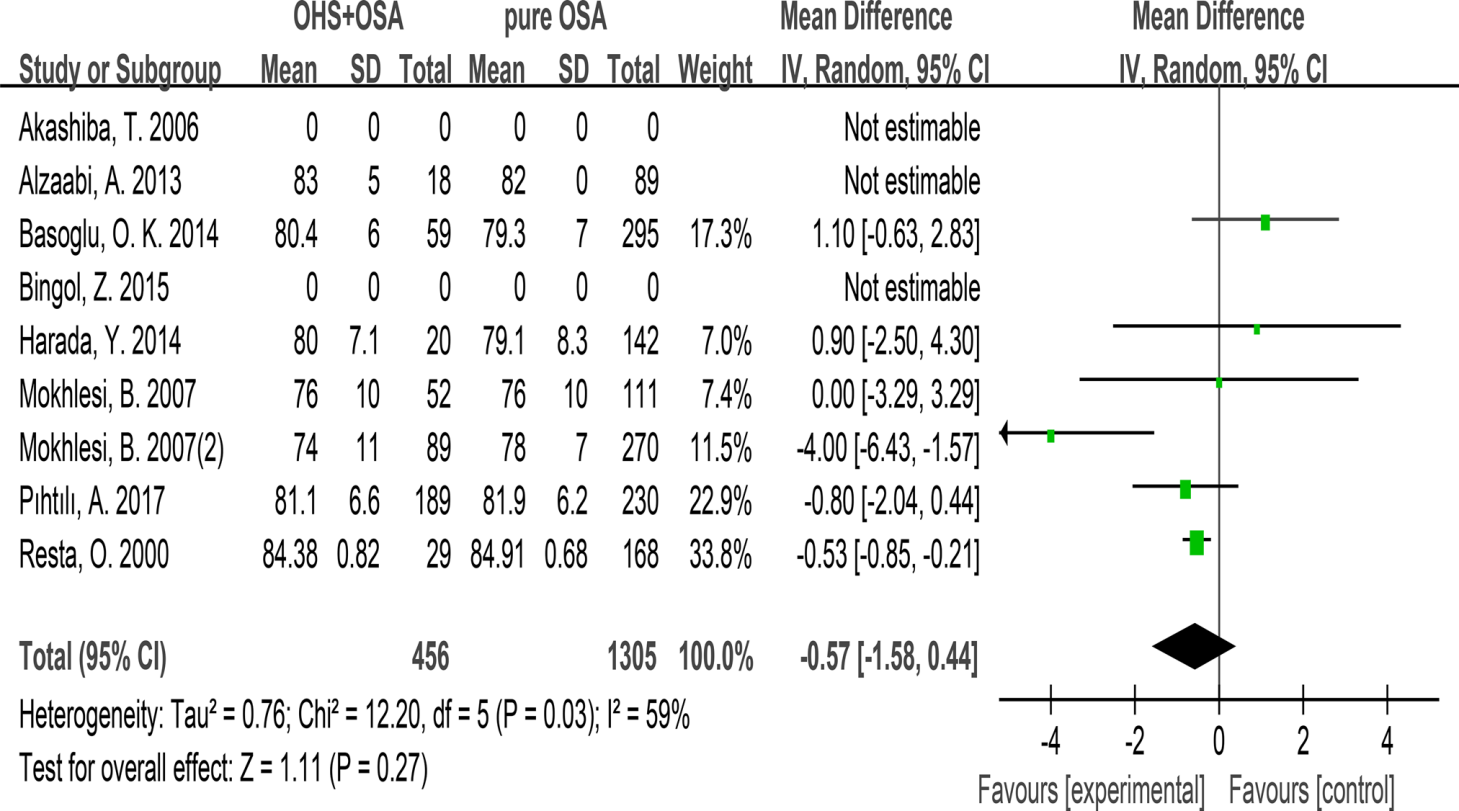
Forest plots of the association between FEV1/FVC and OHS

7 studies evaluated the association between FEV1/FVC and OHS. These studies included 456 patients in the OHS group and 1305 patients in the pure OSA group. FEV1/FVC was not different between the two groups. (MD:–0.57; 95% CI: −1.58 to 0.44; *p* = 0.27) (Figure 4).

### FEV1

**Figure 5:**
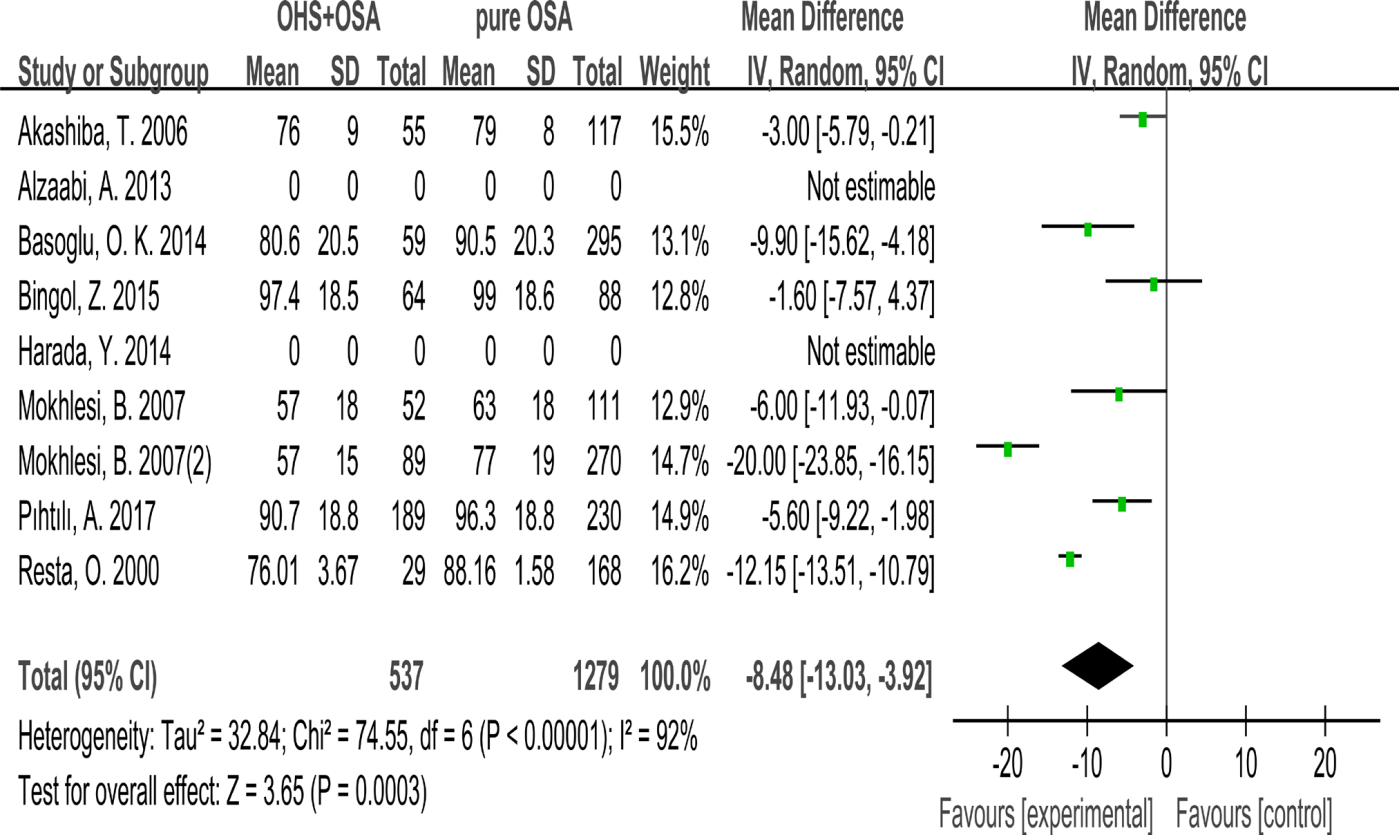
Forest plots of the association between FEV1 and OHS

7 studies evaluated the association between FEV1 and OHS. These studies included 537 patients in the OHS group and 1279 patients in the pure OSA group. FEV1 was significantly different between the two groups. (MD:–8.48; 95% CI: –13.03 to –3.22; *p* = 0.0003) (Figure 5).

### VC%

**Figure 6:**
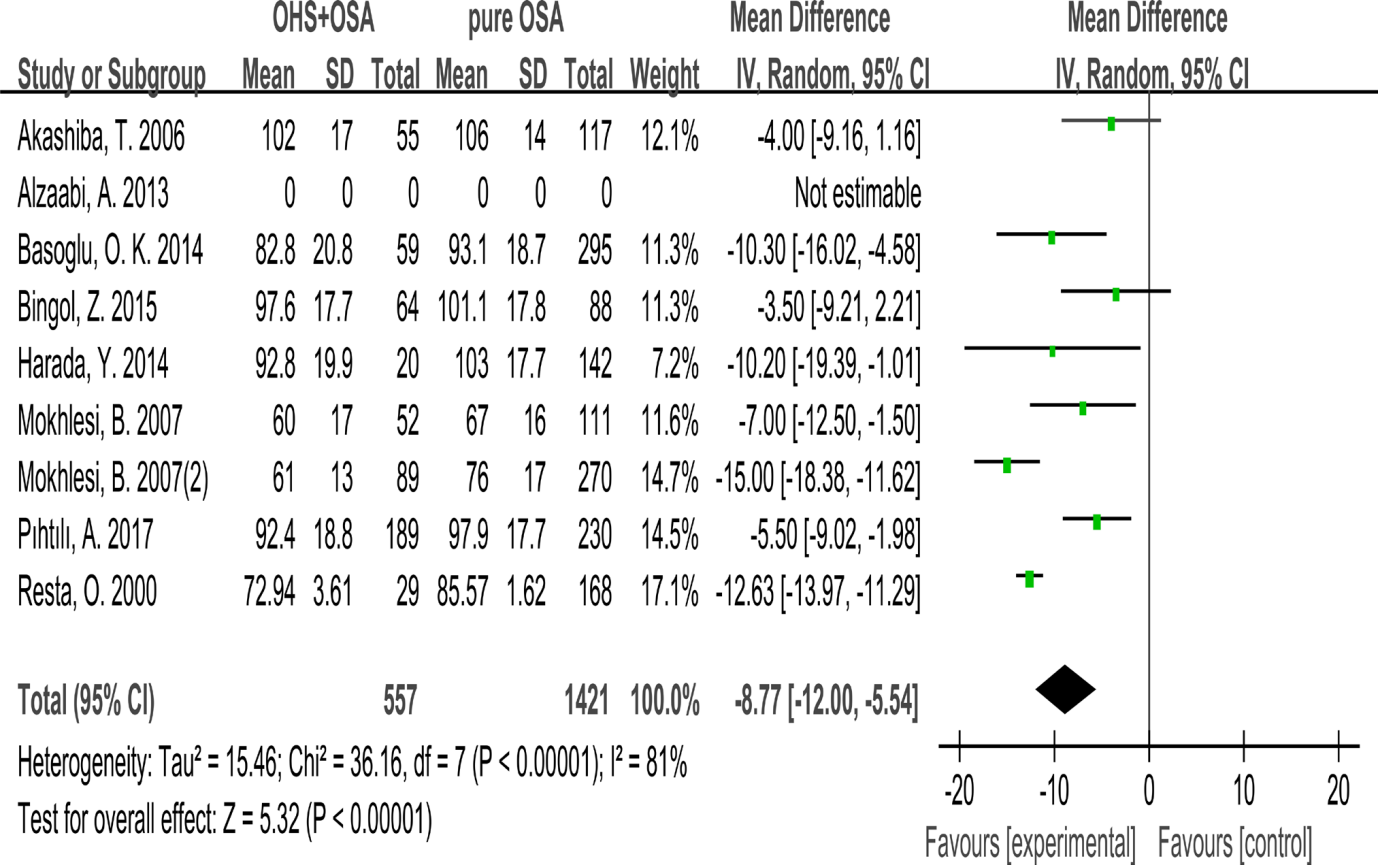
Forest plots of the association between VC% and OHS

7 studies evaluated the association between VC% and OHS. These studies included 557 patients in the OHS group and 1421 patients in the pure OSA group. VC% was significant lower in OHS groups. (MD:–8.77; 95% CI: –12.00 to –5.54; *p* < 0.00001) (Figure 6).

### AHI

**Figure 7:**
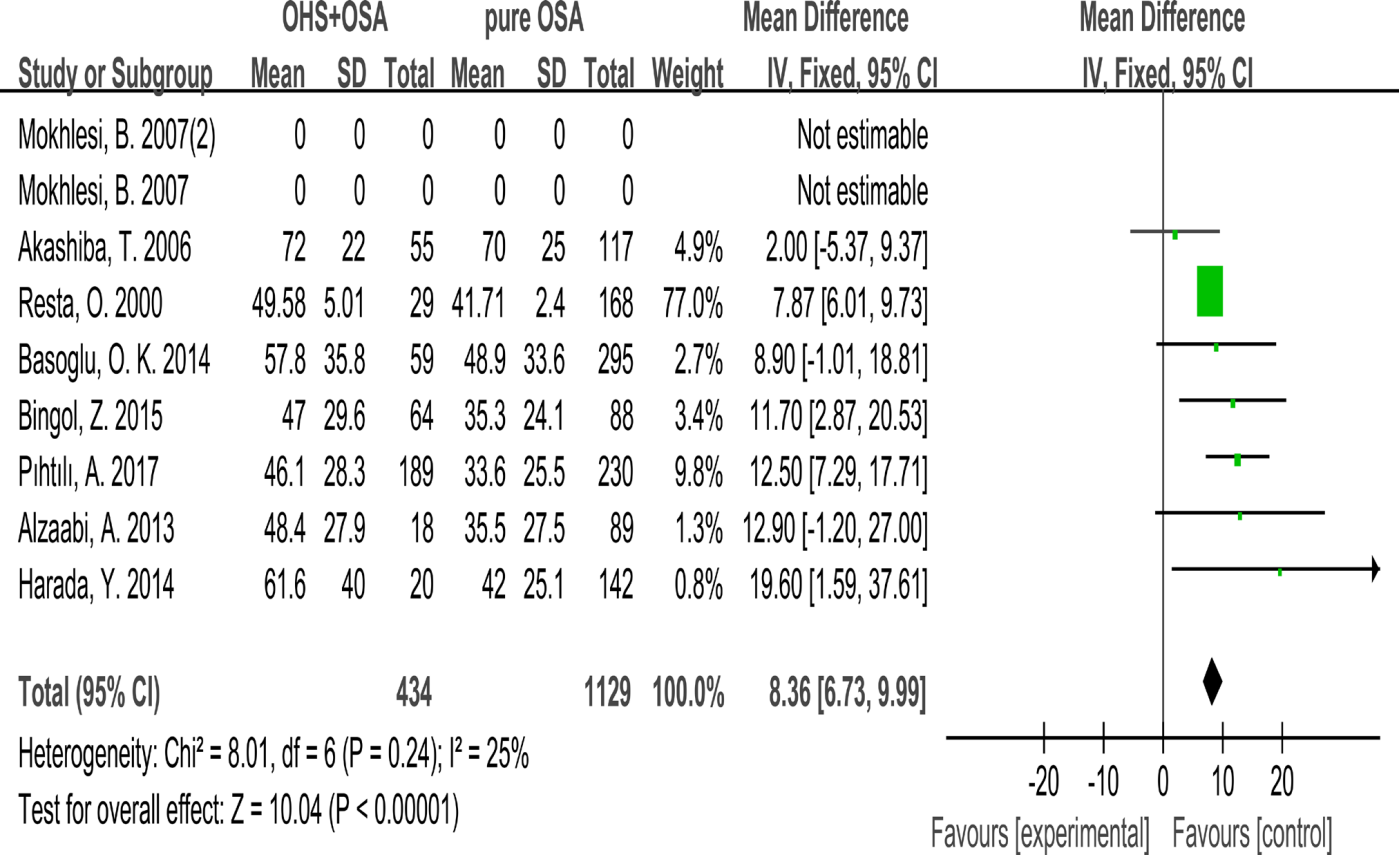
Forest plots of the association between AHI and OHS

Except one study (prospective and retrospective study) from America,7 studies reported AHI for the 1563 included patients, which was significantly more in the OHS group than the pure OSA group. (MD: 8.36; 95% CI: 6.73 to 9.99; *p* < 0.00001) (Figure 7).

### Mean SpO_2_

**Figure 8:**
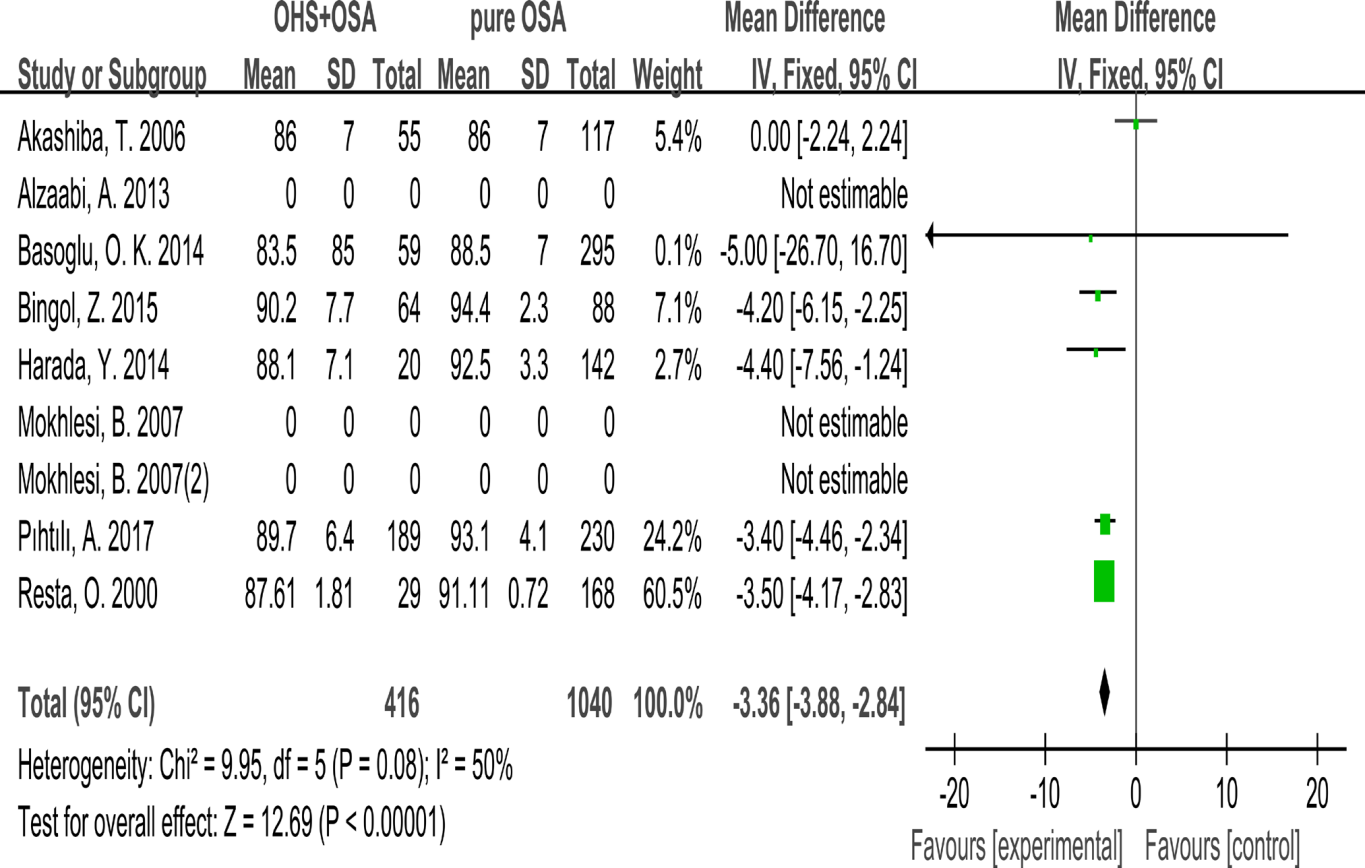
Forest plots of the association between Mean SpO_2_ and OHS

6 studies recorded Mean SpO_2_ during PSG. Finally, it was significantly lower in the OHS group than the pure OSA group. (MD:−3.36; 95% CI: −3.88 to −2.84; *p* < 0.00001) (Figure 8).

### Subgroup analysis

When we sub grouped the patients by BMI level, type of study and race, the associations between OHS and risk factors did not change. For instantly, OHS patient had significantly higher AHI than pure OSA patients in four studies [19, 21, 23–25] with BMI > 30 kg/m^2^ (MD = 10.14; 95% CI = 6.52 to 13.77; *P* < 0.00001). This significant association was also found in three studies [19, 23, 25] with unclear/broad BMI (MD = 8.14; 95% CI = 6.36 to 9.91; *P* < 0.00001). In four studies with cohort studies, OHS patients had significantly lower FEV1% (MD = −1.27, 95% CI = −3.68 to 1.14; *P* = 0.30). Similarly, this difference was also significant in three case-control studies (MD = −0,01, 95% CI = −1.33 to 1.30; *P* = 0.98).

## DISCUSSION

Our meta-analysis confirmed that in obese patients without evidence of obstructive airways disease, Obesity Hypoventilation Syndrome was associated with the following factors: the severity of OSA (as measured by AHI or the degree of nocturnal hypoxemia); BMI; and the degree of restrictive chest wall mechanics. More importantly, the severity of OSA and the impairment of respiratory system mechanics were closely associated with the occurrence of OHS. Specifically, lower FEV1, vital capacity(VC), mean SpO_2_, and higher AHI were found to be some relevant factors.

### Clinical relevance

OHS is one of diseases resulted from obesity, and has close relationship with obesity. It can lead to severe hypoxemia and hypercapnia [26] and other complications. Compared with Arrhythmia, Heart Failure or other Metabolic syndrome resulted from obesity, hypoventilation caused by OHS is easily ignored by clinical doctors. Furthermore, it was very similar to OSAHS in some clinical symptoms. For instance, daytime sleepiness, snoring at night were two similar symptoms.

Pillar G et al. found that obesity itself can increase the risk of developing OSA, and OSA patients could also promote a further increase in the weight of patients [27]. Obesity has influence on both hypoventilation and apnea. OHS and OSA always coexist. Clinic doctors may misdiagnose OHS as OSA because OHS patients also have the same symptoms as OSA patients do, such as night snoring, daytime sleepiness and breathing sleep disorder. But the breathing disorder for OHS patients is mainly performed as hypoventilation and daytime hypercapnia which OSA patients do not have. We observed patients who have both OHS and OSA have higher daytime PaCO^2^ and occupy an obvious proportion in respiratory disorder than pure OSA patients. There were significant differences between OHS patients and OSA patients. OHS patients incline to combine some severe complications, such as chronic respiratory failure, pulmonary hypertension, enlarged right ventricular, which makes worse prognosis and more complicated treatment [28–30]. Moreover, endocrine disease and hypertension are also common complications for OHS patients. With the aggravation of the patients’ condition, the complications merge more easily. Meanwhile, OSA had a great impact on hypertension. Alabri M.A. et al. reported that 20% –45% hypertension patients acquired OHS or OSA [29].

### Fat distribution

Obesity could have a wider effect on OHS patients than OSA patients. OSA patients who were also diagnosed with OHS always tend to show central obesity obviously. Compared with pure OSA patients, they are prone to have greater neck circumstance and waist/hip ratio. The greater the neck circumstance is, the easier the upper airway may collapse. Meanwhile, the increase of waist/hip ratio is regarded as a predictor of the decrease of lung ventilation function [31]. Excessive fat accumulation reduces the compliance of the chest wall and lung, and also leads to a reduction in the efficiency of the diaphragm, which makes FEV1, MVV, VC decrease obviously. The patient's restrictive ventilatory disorder makes the small bronchi and bronchioles of the lungs more likely to collapse and increase the residual volume, resulting in iPEEP. Making a contrast to pure OSA patients’ respiratory muscles, the muscles of patients who were also diagnosed with OHS needs to afford more. The upper airway collapses easily whether OHS patients are taking the sitting and supine position, whereas only supine position can make OSA patients’ upper airway collapses [32]. This is why the patients diagnosed with both OSA and OHS have hypoxia and hypercapnia at daytime. In this meta-analysis, we found neck circumference was significantly greater in the OHS group than the pure OSA group (MD:1.01; 95% CI: 0.10 to 1.92; *p* = 0.03) – which was the same to other studies. Also we found that OHS group had higher BMI (MD:4.72 kg/m^2^; 95% CI: 4.26 to 5.17; *p* < 0.00001). But in our analysis, only two studies reported waist/hip ratio. Finally, we made the conclusion that the Waist/hip ratio was not different between the OHS group and the pure OSA group (MD:–0.04; 95% CI: –0.19 to 0.12; *p* = 0.64). (Figure 7) But Schafer H. and Vladimir M. Macavei, M.D. et al found that Waist/hip ratio of the patients with OHS is greater than normocapnic obese patients [28, 33]. Maybe it is because the subjects we included were not enough for this issue to explain the relationship between OHS patients and pure OSA patients. As a result, we drew an opposite conclusion compared with theoretical results. We should treat this research with a cautious attitude. It still needs lots of relevant studies to have a reliable conclusion.

### Pulmonary function

In this systematic analysis, we found that no significant difference could be found between OHS patients and pure OSA patients on FEV1/FVC. But in aspect of FEV1, OHS patients was different from pure OSA patients. Vladimir M. Macavei, M.D. et al. [33] found that those people who had both OHS and OSA were not different from healthy people on the indicator of FEV1/FVC. But for FEV1,MVV,VC, they can be 80 %, 70% and 70% of healthy people respectively. These indicators would decline further when the people were sleeping or taking supine position. The accumulation of chest and abdomen fat is more serious for OHS patients than pure OSA patients. And the indicator of FEV1/FVC can be always Normal while FEV1, MVV, VC declined, which illustrates that hypoventilation in OHS patients is relevant with abnormal breathing. In conclusion, OSA patients with OHS had restrictive ventilatory disorder, but no significant obstructive ventilatory dysfunction. Via this meta-analysis, we could know that Pulmonary Function Test on pure OSA patients did not have an obvious predicative meaning for patient's condition.

### Polysomnography

Patients with OHS had hypercapnia during daytime awake (hypercapnia was further aggravated during sleep), whereas the presence of daytime hypercapnia was relatively rare in OSA patients. PSG prompted low rates of hypopnea and low oxygen saturation in OSA with OHS patients. All these studies show that not only respiratory ventilation dysfunction is the cause of OHS, but also respiratory center dysregulation was also the cause [34]. On the one hand, carbon dioxide accumulation leads to hypercapnia as chronic restrictive ventilatory disorder reduces ventilation. On the other hand, OSA with OHS patients who were performed with central obesity always had bigger lungs’ elastic resistance and weaker central respiratory drive. With increased elasticity of lung respiration resistance, while leading to lack of central respiratory drive. OHS will not only affect people under sleep in the respiratory function, but also lead to lack of ventilation during the day. The most important pathophysiological changes in OSA patients with OHS are hypoxemia and hypercapnia. With the increase of AHI, oxygen saturation at night will drop more significantly. Hypoxia can excite the sympathetic nerve and increase angiotensin, and then lead to fluctuations in blood pressure. Finally, all these results enhance the role of respiratory depression [35].

In summary, OSA patients are prone to acquire OHS and they are easily ignored. These patients have more serious clinical symptoms and complications than patients with OSA alone. Compared with OSA, OHS has hypoxemia and hypercapnia not only at night but also in daytime. This means OHS patients need to receive a comprehensive treatment. Positive airway pressure (PAP) is considered the first choice for OHS treatment, which is similar to the treatment of OSA. PAP includes continuous positive airway pressure(CPAP) and bi-level positive airway pressure (BiPAP). CPAP is mainly used for the treatment of OSA patients. BiPAP and CPAP have similar functions, but BiPAP was significantly better than CPAP in improving OHS patients’ hypoxemia and tolerance to ventilator. Chau EH el at. [36] indicated that short-term use of PAP (3 weeks) could improve lung gas exchange and sleep-related disordered breathing in OHS patients, and PAP therapy greater than 4 weeks could effectively increase lung capacity and sensitivity of respiratory center to hypercapnia. PAP therapy can reduce OHS mortality. Meanthile, oxygen therapy is thought to be a beneficial treatment for OHS patients [37]. A number of small-scale investigative studies confirmed that disease-related mortality of OHS patients without intervention was significantly higher than obese patients without OHS [38, 39]. Therefore, it is important and necessary to identify OHS patients early and take appropriate interventions to improve prognosis of patients with OHS and their quality of life.

### Limitation

Although our meta-analysis did draw a reliable relationship between OHS, measures of chest wall restriction, and clinical indicators, we were limited by the degree of heterogeneity in our analysis. Firstly, the area where the 9 studies were done is scattered so we could not rule out geographical differences. Secondly, only 5 studies [19, 21, 23–25] included in the meta-analysis reported the mean BMI > 30 kg/m^2^, one study even did not report the mean BMI. 4 of the studies [20, 22, 25] purposefully excluded patients with COPD and the exact percentage of COPD in these studies remains unclear. Various cutoffs of AHI were used in the studies to define OSA. Six studies [20–24] defined OSA by using an AHI > 5 (23% with OHS; 302 of 1296 patients); one studies [18] used an AHI > 10 (15% with OHS; 29 of 197 patients); and one study [19] used an AHI > 20 to define OSA(32% with hypercapnia; 55 of 172 patients). One study (23) did not report an AHI cutoff for defining OSA. Thirdly, even though AHI and impairments in the respiratory system mechanics have been demonstrated as being relevant to the occurrence of OHS, other factors may also be involved in its development in OSA patients who do not have obstructive airways disease. These factors could include anthropometric characteristics, neurohormonal abnormalities such as leptin resistance, or genetic differences [40, 41]. Our meta-analysis did not include the indicators mentioned above as one of the possible influence factors for the development of OHS in OSA patients.

## CONCLUSIONS

In conclusion, our meta-analysis demonstrated three points. Firstly, patients with OSA alone had nocturnal apnea and hypoxia while AHI and Mean SpO_2_ decreased in OSA patients with OHS more significantly. Secondly, it is common for OSA and OHS patients to have fat accumulation while the neck circumference is greater in OSA patients with OHS. Thirdly, the indicator of FVC and VC decreased significantly in OSA patients with OHS while FEV1/FVC remained the same as pure OSA patients, which illustrated that these patients’ ventilation is based on restricted ventilatory disorder. This meta-analysis contributed compelling evidence that the indicators mentioned above may be a useful marker in predicting OHS in OSA patients. Nevertheless, the inherent limitations of included studies stop us from reaching definitive conclusions despite our rigorous methodology. We look forward to large-volume, well-designed studies to update and confirm the findings of this analysis.

## SUPPLEMENTARY MATERIALS TABLES


