# Supplementary material for: The relationship between obstructive sleep apnea and obesity hypoventilation syndrome: a systematic review and meta-analysis
Source: Oncotarget. 2017 Oct 3;8(54):93168–78. doi: 10.18632/oncotarget.21450 (PMC5696252; doi:10.18632/oncotarget.21450)

The relationship between obstructive sleep apnea and obesity hypoventilation syndrome: a systematic review and meta-analysis

SUPPLEMENTARY MATERIALS

Supplementary Tables 1: Evaluation of the quality of the 10 included studies by using the Newcastle-Ottawa Scale#

Factor	Study type	Selection				Comparability	Exposure or outcome			Total
		1	2	3	4		1	2	3	
Resta et al., 2000	Cohort Study	★	★	★	★		★			5
Akashiba et al., 2006	Cohort Study	★	★	★	★	★	★			6
Mokhlesi et al., 2007	Case-Control Study	★	★	★	★		★	★	★	7
Mokhlesi et al., 2007 (2)	Cohort Study	★	★	★	★		★			5
Alzaabi et al., 2013	Cohort Study	★	★	★	★	★	★			6
Basoglu et al., 2014	Case-Control Study	★	★	★	★	★	★	★	★	8
Harada et al., 2014	Cohort Study	★	★	★	★	★	★			6
Bingol et al., 2015	Cohort Study	★	★	★	★	★	★			6
Pıhtılı et al., 2017	Case-Control Study	★	★	★	★	★	★	★	★	8

The Newcastle-Ottawa Scale criteria are listed in supplemental files.

Supplementary Tables 2: Weighted means and percentages of patients with and without OHS per risk factor

outcomes	Studies, no.	OHS+OSA	Pure OSA	WMD/OR* (95% CI)	*P* value	Study heterogeneity I2
BMI	9	575	1510	4.72 (4.26, 5.17)	0.001	90%
AHI	9	434	1129	8.36 (6.73, 9.99)	< 0.00001	25%
Age	9	575	1510	0.08 (−0.42, 0.25)	0.62	90%
Mean SpO^2	6	416	1040	−3.36 (-3.88, −2.84)	< 0.00001	50%
FEV1	7	537	1279	−8.48 (−13.03, −3.22)	0.0003	92%
VC%	8	557	1421	−8.77 (−12.00, -5.54)	< 0.00001	81%
FEV1/FVC	7	456	1305	−0.57 (−1.58, 0.44)	0.27	59%
Neck circumference	4	172	693	1.01 (0.10, 1.92)	0.03	59%
Waist/hip ratio	2	84	230	−0.04 (−0.19, 0.12)	0.64	46%
Gender (male)	8	529	1406	1.62* (0.66, 3.98)	0.29	93%

Supplementary Tables 3: Characteristics of included studies

Study	Year	Country	Patient no.		BMI > 30 Kg/m²	COPD	Matching
			OHS+OSA	OSA			
Resta et al.[18]	2000	Italy	29	168*	No	?	1, 2, 3, 4, 5, 6, 7, 8, 9, 11
Akashiba et al.[19]	2006	Japan	55	117₰	Yes	?	1, 2, 3, 4, 6, 7
Mokhlesi et al.[20]	2007	America	52	111₱	No	excluded	1, 3, 6, 7, 8, 11
Mkhlesi et al.[20]	2007 (2)	America	89	270₱	No	excluded	1, 2, 3, 6, 7, 8, 11
Alzaabi et al.[21]	2013	UAE	18	89₱	Yes	?	1, 2, 3, 8, 11
Basoglu et al.[22]	2014	Turkey	59	295₱	?	excluded	1, 2, 3, 4, 5, 6, 7, 8, 9, 11
Harada et al.[23]	2014	Japan	20	142₱	Yes	?	1, 2, 3, 4, 5, 7, 8, 9, 10, 11
Bingol et al.[2]4	2015	Turkey	64	88₱	Yes	?	1, 2, 3, 4, 6, 7, 9, 10, 11
Pıhtılı et al.[25]	2017	Turkey	189	230?	Yes	excluded	1, 2, 3, 4, 5, 6, 7, 8, 11

UAE = United Arab Emirates; 1 = BMI; 2=AHI; 3 = Age; 4 = mean SpO2; 5=TST SpO2; 6 = FEV1; 7 = VC%; 8 = FEV1/FVC%; 9 = Neck circumstance; 10 = Waist/hip ratio; 11 = Gender. ? = not reported, * = OSA defined as AHI > 10, ₰ = OSA defined as AHI > 20, ₱ = OSA defined as AHI > 5.

Supplementary Tables 4: Search strategy profile

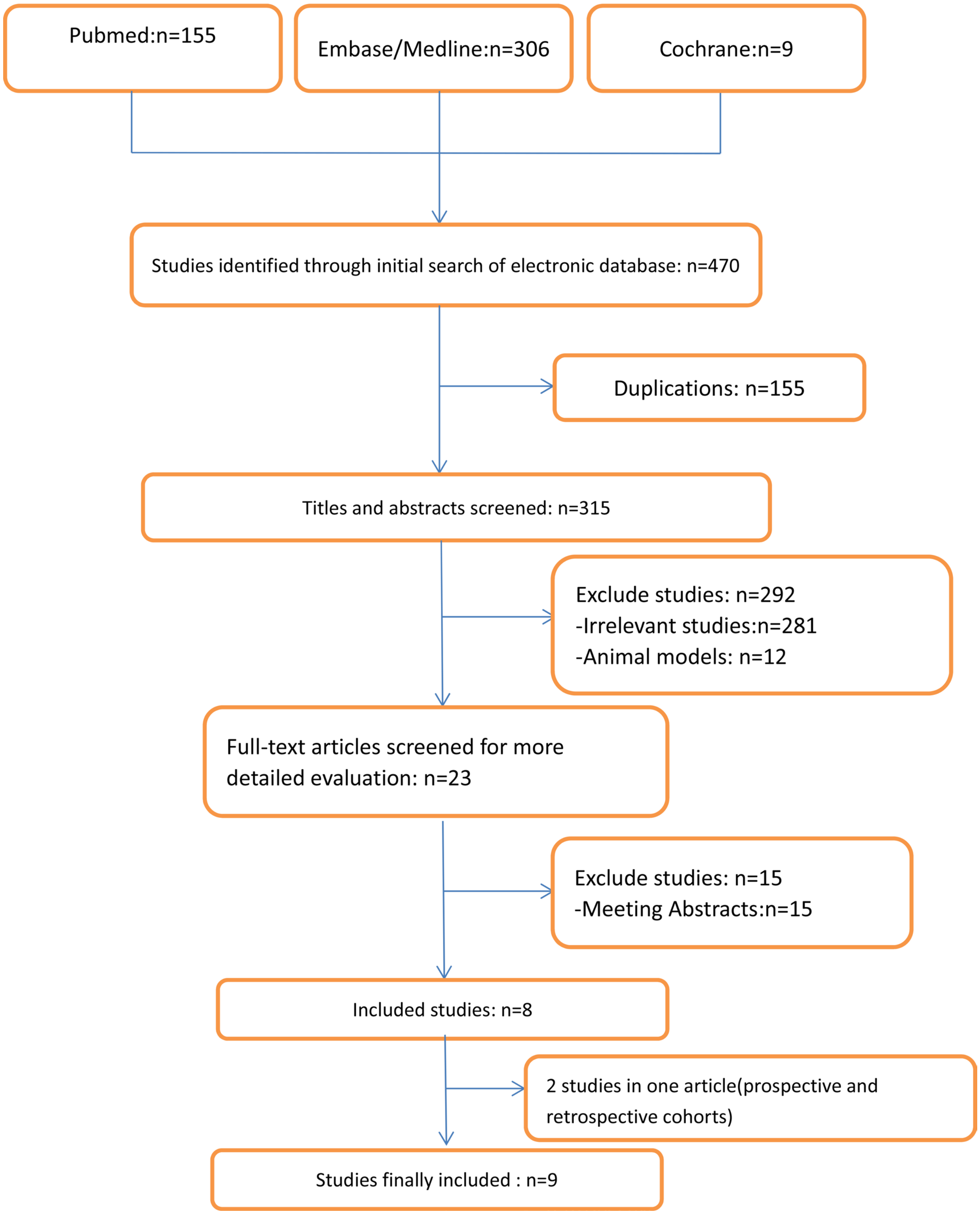

Supplement: Supplementary file 1 [file oncotarget-08-93168-s001.pdf]
